# rTMS Over Dorsolateral Prefrontal Cortex Augments Dual‐Task Training for Mobility, Balance and Cognition in Sub‐Acute Stroke: A Randomized Controlled Trial

**DOI:** 10.1002/pri.70259

**Published:** 2026-06-24

**Authors:** Lei Yang, Xiaoying Lin, Jingyi Lu, Xi Chen, Liuyan Wang, Hui Yang, Ying Gao, Marco Yiu Chung Pang

**Affiliations:** ^1^ Department of Rehabilitation Medicine The Second People's Hospital of Kunming Kunming Yunnan China; ^2^ Department of Rehabilitation Medicine The First Affiliated Hospital Xi'an Jiaotong University Xi'an China; ^3^ Department of Rehabilitation Sciences The Hong Kong Polytechnic University Hung Hom Hong Kong China

**Keywords:** cognition, dual‐task, randomized controlled trial, stroke, transcranial magnetic stimulation, walking

## Abstract

**Objective:**

To evaluate the effects of adding repetitive transcranial magnetic stimulation (rTMS) to dorsolateral prefrontal cortex (DLPFC) with dual‐task (DT) exercise on DT mobility, balance and cognitive performance in individuals with sub‐acute stroke.

**Methods:**

Thirty sub‐acute stroke patients [age, mean (SD) = 59.2 (7.9) years] were randomly assigned to the experimental group (*N* = 15), or the sham stimulation group (*N* = 15). 5 Hz rTMS (90% resting motor threshold, 1200 pulses/session) or sham stimulation was applied to the ipsilesional DLPFC, 1 session/day, 5 days/week for 2 weeks, followed by dual‐task training. Two mobility tests [10 m walking and timed‐up‐and‐go (TUG) test] and two cognitive tasks (serial 3 subtractions and verbal fluency) were assessed separately [that is, single‐task (ST) condition] and concurrently (i.e., DT condition) before and after the intervention.

**Results:**

The experimental group had greater improvement in TUG time under both ST and DT conditions (*p* < 0.05), while the step length of the paretic leg during the 10 m walking test of the experimental group only showed better improvement under ST condition and in DT condition when performed with serial 3 subtractions task. Cadence changes did not reach significance in any DT condition. Greater increment in the DT cognitive performance was observed only when verbal fluency task performed with 10 m walking test in the experimental group. The experimental group had better gain in Montreal cognitive assessment, Mini balance evaluation systems test, and Activities‐specific balance confidence scores than controls.

**Discussion:**

The 2‐week rTMS to DLPFC combined with DT training augments DT walking performance, and improves balance and cognitive function in mild to moderate motor and cognitive impairment individuals with sub‐acute stroke.

**Implications of Physiotherapy Practice:**

This combined intervention is a feasible, effective strategy to improve real‐world mobility in sub‐acute stroke rehabilitation.

**Trial Registration:**

Chinese Clinical Trial Registry platform (www.chictr.org.cn), with the registration number ChiCTR2200066237

## 1 Introduction

Functional ambulation necessitates the ability of dividing attention, such as walking when talking, and walking when holding a cup of water. In these scenarios, two tasks are performed simultaneously [that is, dual‐task (DT) conditions]. Compared with performing a single task alone [that is, single‐task (ST) condition], performing two tasks simultaneously may lead to degradation in performance in one or both component tasks (i.e., cognitive‐motor interference) (Plummer et al. 2013).

Impairment in walking function is very often a major concern in stroke rehabilitation (Gilmore et al. 2021). Some common gait‐related problems observed in individuals with stroke include slower gait speed, abnormal movement patterns, poorer adaptive function (e.g., obstacle avoidance), and greater susceptibility to falls (Gilmore et al. 2021; McCulloch et al. 2010). More recently, DT mobility function after stroke has garnered increasing attention in research and clinical practice (Tsang et al. 2022; He et al. 2018). Mounting evidence has suggested that individuals with stroke tend to show more deterioration in performance of either the cognitive, mobility, or both tasks under DT condition, when compared with age‐matched healthy older adults (Yang et al. 2018; Al‐Yahya et al. 2011). Such compromised DT ability post‐stroke would hinder the optimal functioning in “real‐life” scenarios (Plummer et al. 2013; Plummer and Eskes 2015). DT mobility function has also been associated with falls post‐stroke (Tsang et al. 2013, 2022). In order to fully prepare individuals with stroke for returning community after discharge from in‐patient care, improving DT ability should thus be an important goal of stroke rehabilitation (Plummer et al. 2013).

In the past decade, a number of trials have investigated the effect of DT training among individuals with stroke. A systematic review in 2018 suggested that DT training may be beneficial for improving DT balance and mobility performance after stroke (He et al. 2018). However, the results from the reviewed trials were inconsistent and the effect sizes were small (He et al. 2018). Later, in a randomized controlled trial, Pang et al. demonstrated that 8 weeks of DT exercise reduced DT interference during forward walking (combined with verbal fluency or with serial‐3‐subtractions) and timed‐up‐and‐go test (combined with verbal fluency) (Pang et al. 2018). A study by Plummer et al. involving 26 participants with stroke showed that although DT exercise group had overall no significant effect on cognitive‐motor interference compared with the ST exercise group, their subgroup analysis revealed that those with greater interference at baseline may benefit more from DT (Plummer et al. 2022). In a randomized controlled study, Baek et al. demonstrated that a 6‐week DT treadmill gait training induced greater reduction of DT interference in all gait parameters measured (e.g., speed, cadence, variability) and cognitive performance in people with chronic stroke, when compared with ST treadmill gait training (Baek et al. 2021). Hereafter, increasing research efforts have been directed toward identifying alternative strategies to further enhance DT mobility function post‐stroke.

Non‐invasive brain stimulation (NIBS), such as repetitive transcranial magnetic stimulation (rTMS), has been widely used in rehabilitation for depression, cognitive function, upper limb motor function, balance and gait after stroke (Hara et al. 2021; Zhang et al. 2017; Behrangrad et al. 2021; Xie et al. 2021). Several functional near infrared spectroscopy (fNIRS) studies demonstrated that dorsolateral prefrontal cortex (DLPFC) was strongly associated with executive function and higher order cognitive function, leading to the postulation that DLPFC may play a critical role in DT walking, making it a potential target for intervention (Beurskens et al. 2014; Fraser et al. 2016). Several studies used transcranial direct current stimulation (tDCS) to up‐regulate the excitability of DLPFC and found that DT gait performance was improved in Parkinson's disease and older adults with limited mobility (Lattari et al. 2017; Swank et al. 2016). It is thus possible that adding rTMS on DLPFC to a DT training program may confer additional benefits on DT walking function. Only one previous trial investigated the effect of rTMS on DT walking among individuals with chronic stroke and found no significant improvement in DT walking speed, possibly due to insufficient dosing (single session), lack of concurrent DT training, and use of a site‐control (M1 stimulation) rather than a sham‐control (Goh et al. 2020). Moreover, whether combining DT training with rTMS on DLPFC would augment the training benefits for individuals with stroke is still unknown.

Our study addresses these gaps by combining multi‐session rTMS (10 sessions) with structured DT exercise in sub‐acute stroke, using a double‐blind sham‐controlled design. This randomized controlled study aimed to evaluate the effects of combined DT exercise with rTMS to DLPFC on DT mobility, balance, and cognitive performance in individuals with sub‐acute stroke. We hypothesized that real rTMS + DT training would lead to greater improvements in DT walking performance, balance, and cognitive function compared to sham rTMS + DT training.

## 2 Methods

### 2.1 Ethical Approval, Informed Consent and Trial Registration

The trial was approved by the Ethics Committee of the Second People's Hospital of Kunming, and was conducted in accordance with the Declaration of Helsinki. The trial was registered in the Chinese Clinical Trial Registry (no. ChiCTRC2200066237) and reported according to the CONSORT reporting guidelines (Schulz et al. 2010). All participants provided written informed consent before data collection.

### 2.2 Inclusion and Exclusion Criteria

Participants were recruited from the Second People's Hospital of Kunming. The interventions were performed in the physical therapy room of the rehabilitation department, while the assessments were performed in the laboratory of the rehabilitation department. The inclusion criteria were: (1) a diagnosis of unilateral stroke; (2) aged between 30 and 80 years; (3) first onset and during 1–6 months; (4) medically stable; (5) having the ability of walking 10 m independently; (6) able to follow 3‐step commands. Exclusion criteria were as follows: (1) other neurological conditions (e.g., Parkinson's disease, brain injury); (2) cerebellar or brainstem injury; (3) contraindications to TMS, such as wearing a cardiac pacemaker, intracranial metal implants or skull defect, a previous history of epilepsy, pregnancy, etc; (4) severe cognitive (Montreal Cognitive Assessment score < 21) or speech impairment (significant receptive or expressive aphasia); (5) other serious illnesses that influenced functional performance.

### 2.3 Study Design

This was a double‐blinded, randomized placebo controlled clinical trial. The study investigator used the Excel software to generate the randomization sequence. Treatment allocation was concealed in an opaque envelope from the study investigator and kept closed until the baseline assessment was completed by a blinded assessor. After the baseline assessment, all participants were randomly assigned to the experimental group or the control group. Participants were blinded to the rTMS condition (real rTMS or sham rTMS), and were told that “the sound produced by the rTMS device was very modest, and the cutaneous sensation induced by rTMS was very little”.

### 2.4 Sample Size Estimation

G*Power 3.1 software (Heinrich‐Heine‐Universitat, Dusseldorf, Germany) was used to estimate the sample size. A pilot study by Goh et al. showed that stimulation of DLPFC resulted in greater improvement in DT gait speed than stimulation of the primary motor cortex or supplementary motor area, with a partial eta‐squared (*η*_*p*_) (Gilmore et al. 2021) value of 0.18 (equivalent to *f* = 0.46). A more conservative estimate was made here because we compared real rTMS stimulation with sham stimulation. Given the short intervention duration (2 weeks), a 10% attrition rate would be more realistic; however, we conservatively estimated 20% to ensure adequate power. Based on 2 × 2 analysis of variance (ANOVA), the assumption of a medium effect size (*f* = 0.3), an *α* of 0.05, and a power of 0.80, the estimated sample size was 30 participants in total.

### 2.5 Intervention Protocol

Before performing the DT exercise in each intervention session, the experimental group received the real rTMS, while the control group received the sham rTMS. Both groups received their respective interventions one session/day, 5 days/week, for 2 consecutive weeks (i.e., 10 sessions in total).

Real rTMS: Participants were placed in a supine position on the treatment bed. Firstly, an experienced physical therapist identified the location of DLPFC on the affected hemisphere by the surface anatomy, which was approximately the projection to the skull of Brodmann area 9/46, 5 cm forward along the parasagittal line at the M1 cortex location (Kim et al. 2022). Next, we observed the contraction of contralateral abductor pollicis brevis to determine the resting motor threshold (RMT) (Goh et al. 2020). Then, the therapist performed 5 Hz rTMS to the affected hemisphere DLPFC at 90% of RMT using a 90 mm annular coil connected to a magnetic stimulator (Weisi Company, Nanjing, China). The rTMS pulses in 10‐s trains with an intertrain interval of 30 s were delivered. Therefore, each participant received a total of 1200 rTMS pulses in 16 min.

Sham rTMS: The stimulation intensity of the control group was adjusted to 10% RMT so that the magnetic flux was too weak to cross the bones to reach the DLPFC, while the other parameters were the same as the experimental group.

DT exercise: Participants in both groups received the same DT balance and mobility training after each rTMS stimulation session. The exercise protocol was described in details elsewhere (Pang et al. 2018). Basically, the motor tasks involved mobility, balance, and agility training station, while the cognitive tasks involved a wide range of activities, such as naming objects, carrying out a conversation, remembering numbers, etc. Participants were asked to perform the two tasks simultaneously in random order by other qualified physical therapists.

### 2.6 Outcome Measurements

All demographic information (e.g., age, gender and hemiparetic side, etc.) were collected at baseline assessment session. All outcome measurements were performed on two occasions, first at baseline and again within 1 week after the completion of intervention.

#### 2.6.1 Primary Outcomes

The walking speed in 10 m walking test and the time taken to complete timed up‐and‐go (TUG) test under DT condition were the primary outcomes.

Ten *m* walking test: A computerized system (GAITRite, NJ, USA) was used to collect data on gait parameters (speed, step length, and cadence). Participants were asked to walk along a 14 m walkway at a comfortable speed (i.e., ST condition). Only the gait data collected in the middle 10 m of the walkway were used for analysis.

TUG test: Participants were encouraged to stand up from the chair as quickly as possible, walk forward for 3 m, turn around, return to the chair, and then sit down. The time taken to complete the TUG test was recorded and used for subsequent analysis.

Next, one of the two cognitive tasks (serial 3 subtraction or verbal fluency) was added while performing the mobility test (i.e., DT condition). For the serial 3 subtraction task, the participants were instructed to subtract 3 continuously from a random number between 0 and 50, as fast and as accurately as possible. For the verbal fluency task, participants were asked to name as many items as possible in a given category, such as animals, clothing, etc. In order to facilitate the comparison across participants before and after the intervention, the same category of items was combined with a given mobility task (i.e., 10 m walking test + animals; TUG test + clothing). However, the sequence of testing was randomized to prevent the learning effect as much as possible. To prevent physical and mental fatigue, only one trial was tested in our assessment, since good to excellent test‐retest reliability of the DT walking was established with single trials (intraclass correlation coefficient, ICC_2,1_ = 0.70–0.93) (Yang et al. 2016). The gait parameters and correct response rate (CRR) were recorded during performance of the DTs.

Lastly, participants were asked to perform the two cognitive tasks while sitting in a stable chair (i.e., ST condition). The time given to perform each cognitive task was matched with the time taken to perform DT walking combined with the same cognitive task. A different starting number was given for the serial 3 subtraction task to prevent learning effect. The correct response rate (CRR) of the cognitive tasks was calculated as follows:

CRR=numberofcorrectresponses÷time

Where a lower CRR value indicated worse performance.

#### 2.6.2 Secondary Outcomes

Balance function: The Mini Balance Evaluation Systems test (Mini‐BESTest) was used to evaluate the balance ability in several dimensions, such as biomechanical constraints, stability limits/verticality, anticipatory postural adjustments, postural responses, sensory orientation and gait stability. It has demonstrated excellent intra‐ (ICC_3,1_ = 0.97) and inter‐rater (ICC_2,1_ = 0.96) reliability (Tsang et al. 2013).

Balance self‐efficacy: The Activities‐specific Balance Confidence (ABC) scale is a questionnaire with 16 items representing basic daily tasks (e.g., walking around the house, up and down the stairs) and more difficult tasks in the community (e.g., walking in a crowded shopping mall (Kim et al. 2022). This scale requires participants to assess their balance confidence on a continuous scale ranging from 0% to 100%. The mean score of the 16 items was used for analysis. Excellent reliability (ICC = 0.85∼0.99) and good convergent validity have been established (Mak et al. 2007).

Global cognitive function: Montreal Cognitive Assessment (MoCA) was used to assess visuospatial executive function, naming, attention, language, abstraction, delayed recall, and orientation. The maximum score is 30. If the participant's education level was less than 6 years, the total score was increased by one point to correct the level of illiteracy (Nasreddine et al. 2005). It has shown excellent reliability (ICC = 0.87∼0.96) and discriminant validity (A. Wong et al. 2009).

Stroop color and word test: Participants were instructed to read three different maps as quickly and correctly as possible, with the evaluator recorded the time they required (Scarpina and Tagini 2017). The Stroop color and word test showed excellent predictive validity for the prediction of dementia (area under the curve ≥ 0.85) and good to excellent test‐retest reliability in healthy young adults (Pearson's *r* = 0.67∼0.83) (Génier Marchand et al. 2017; Franzen et al. 1987).

Trail Making Test A and B (TMT‐A and B): The TMT consists of two parts. The TMT‐A required the participants to draw lines in turn to connect the 25‐surrounded numbers distributed on a piece of paper. The task requirements of TMT‐B were similar, but the participants had to alternate between numbers and letters (e.g., 1, A, 2, B, 3, C, etc.). The fraction of each part represents the time required to complete the TMT. If it exceeded 300s, it was calculated as 300s (Tombaugh 2004). The TMT had excellent reliability (ICC = 0.90∼0.95) and discriminant validity (Park and Scot 2022).

Digit Span Test (DST): The DST consists of two parts (sequential and reverse order) was used to assess working memory ability. The assessor pronounced a series of numbers at a rate of about 1 number per second. The list was then repeated by the subjects in the same order. Next, participants had to backwards align the numbers in the digit‐backward test. The length of the sequence increases in digital spans. Testing started with a double number sequence and then gradually increased to eight sequences. In this study, we determined the maximum digit length obtained for each participant, especially the longest sequence they could answer correctly in both the forward and backward digit span, as scored for the subject (Ryan et al. 1996). The test‐retest reliability correlation coefficients of subtests were 0.66–0.81 (Thammachai et al. 2022).

### 2.7 Statistical Analysis

All data were analyzed using the software SPSS 24.0 (IBM, Armonk, USA). Intention‐to‐treat analysis (ITT) approach was used for data analysis. No participants withdrew from the study; therefore, ITT analysis was equivalent to per‐protocol analysis. Normality of the data was assessed using the Shapiro‐Wilk test. Depending on the level of data, the baseline characteristics were tested by independent *t* tests, Mann‐Whitney U tests, or Chi‐square tests. If the normal distribution of parameter statistics was satisfied, repeated‐measures analysis of variance (ANOVA) would be applied for group × time interaction effect and time effect. Post‐hoc analysis with Bonferroni adjustment were also performed if significant results were found. The effect size was denoted by the partial eta‐squared (*η*_*p*_) (Gilmore et al. 2021). Values of 0.01, 0.06, and 0.14 represent small, medium, and large effect size, respectively. For the between‐group comparison, Hedges' *g* was computed under both ST and DT conditions based on the mean change score/SD of change score with an online calculator, in order to facilitate the observation of intervention effect across parameters (Lenhard and Lenhard n.d.). The value of Hedges' *g* was regarded as small, moderate, and large, if it was 0.2, 0.5, and 0.8, respectively. A significance level of 0.05 was used.

## 3 Results

**FIGURE 1:**
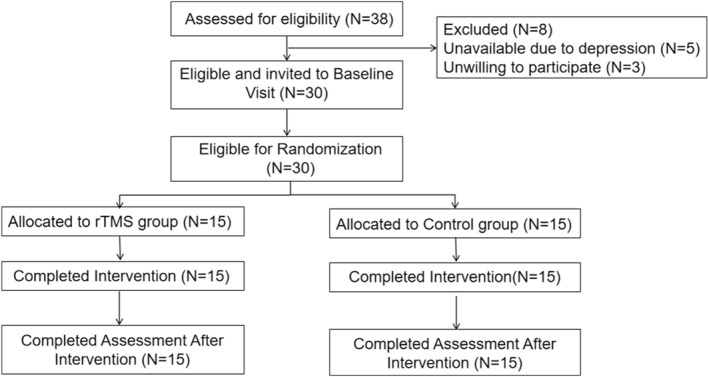
Consort flowchart. A consort flowchart showing participant enrollment, allocation, intervention and analysis.

Thirty out of 38 participants with stroke screened met the eligibility criteria. Details of participant recruitment are shown in Figure 1.

### 3.1 Demographics

The participant characteristics are shown in Table 1. Overall, the mean age was 59.2 ± 7.9 years and the average time since stroke onset was 104 ± 26 days. A total of 25 participants needed walking aid at outdoors. The CMSA score (median and interquartile range) for paretic leg and foot were 5 (5, 6) and 4 (3, 4), respectively, indicating mild impairment in lower limb motor function. At baseline, there were no significant differences in any demographic (Table 1) or outcome variables at baseline (Tables 2, 3, 4) between the two groups (*p* > 0.05).

**TABLE 1:** Characteristics of participants.

	All participants (*n* = 30)	Experimental group (*n* = 15)	Control group (*n* = 15)	*p*‐value^a^
Basic demographics				
Age, y^b^	59.2 ± 7.9	59.0 ± 7.5	59.3 ± 8.6	0.911
Gender, female (*n*, %)^c^	15 (50)	8 (53.3)	7 (46.7)	0.715
Body mass index (kg m^−2^)^b^	22.5 ± 2.4	22.4 ± 2.7	22.6 ± 2.2	0.860
Educational level, y^b^	11.2 ± 3.7	11.13 ± 3.9	11.3 ± 3.6	0.885
Stroke characteristics				
Time since onset (days)^b^	104.4 ± 25.5	103.1 ± 25.1	105.8 ± 26.8	0.775
Hemiparetic side (left/right, *n*)^c^	14/16	8/7	6/9	0.464
CMSA leg score (1–7)^d^	5 (5, 6)	5 (4, 5)	5 (5, 6)	0.161
CMSA foot score (1–7)^d^	4 (3, 4)	4 (3, 4)	4 (4, 5)	0.653
Type (ischemic/hemorrhagic, *n*)^c^	13/17	7/8	6/9	0.713
Use of walking aid outdoors (yes/no, *n*)^c^	25/5	12/3	13/2	0.624
Other characteristics				
Total number of medications (*n*)^b^	0.6 ± 1.1	0.8 ± 1.5	0.5 ± 1.0	0.401
Total number of sessions attended (*n*)^b^	9.2 ± 0.8	9.3 ± 0.7	9.1 ± 0.8	0.640

Abbreviation: CMSA: Chedoke‐McMaster Stroke Assessment.

^a^ *p* values for between‐group comparisons.

^b^ Mean ± SD presented for continuous variables.

^c^ Presented for nominal variables.

^d^ Median (interquartile range) for ordinal variables.

**TABLE 2:** Primary outcomes: walking ability.

	rTMS group			Control group			Effect size	Group × time interaction effect	Time effect
	Pre	Post	Mean change after intervention (95% CI)	Pre	Post	Mean change after intervention (95% CI)	Hedges' *g* (95% CI)	*F*‐value, *p*‐value, *η*_p_^2^‐value	*F*‐value, *p*‐value, *η*_p_^2^‐value
Under ST condition									
Speed (cm/sec)	63.0 ± 14.5	72.7 ± 17.1^a^	9.7 (5.7, 15.5)	64.6 ± 21.0	71.0 ± 22.9^a^	6.5 (4.3, 9.3)	0.41 (−0.32, 1.13)	*F*_1,28_ = 1.257	*F*_1,28_ = 31.334
								*p* = 0.272	*p* < 0.001
								*η*_p_^2^ = 0.043	*η*_p_^2^ = 0.528
TUG	21.1 ± 4.9	16.5 ± 4.1^a^	−4.6 (−6.0, −3.2)^b^	21.3 ± 6.7	19.2 ± 6.7^a^	−2.1 (−2.6, −1.5)	1.21 (0.43, 2.0)	*F*_1,28_ = 10.897	*F*_1,28_ = 79.209
								*p* = 0.003	*p* < 0.001
								*η*_p_^2^ = 0.280	*η*_p_^2^ = 0.739
Step length (affected side, cm)	41.7 ± 4.9	45.5 ± 5.0^a^	3.8 (3.0, 4.7)^b^	41.1 ± 3.3	43.1 ± 3.7^a^	2.0 (1.6, 2.5)	1.36 (0.56, 2.15)	*F*_1,28_ = 13.456	*F*_1,28_ = 142.935
								*p* = 0.001	*p* < 0.001
								*η*_p_^2^ = 0.325	*η*_p_^2^ = 0.836
Cadence (step/min)	88.8 ± 11.2	94.4 ± 13.0^a^	5.6 (4.1, 7.3)^b^	87.9 ± 11.2	89.7 ± 9.7	1.8 (−0.3, 3.8)	1.07 (0.3, 1.83)	*F*_1,28_ = 8.272	*F*_1,28_ = 30.861
								*p* = 0.008	*p* < 0.001
								*η*_p_^2^ = 0.228	*η*_p_^2^ = 0.524
Under DT condition (performed with serial 3 subtraction task)									
Speed (cm/sec)	55.8 ± 13.0	65.4 ± 17.5^a^	9.6 (5.3, 16.5)	57.2 ± 18.7	63.0 ± 20.7^a^	5.8 (4.0, 8.0)	0.27 (−0.45, 1.0)	*F*_1,28_ = 1.533	*F*_1,28_ = 24.261
								*p* = 0.226	*p* < 0.001
								*η*_p_^2^ = 0.052	*η*_p_^2^ = 0.464
TUG (sec)	23.6 ± 3.3	19.4 ± 4.2^a^	−4.2 (−5.1, −3.4)^b^	23.4 ± 5.9	20.8 ± 6.3^a^	−2.6 (−3.5, −1.5)	0.91 (0.16, 1.67)	*F*_1,28_ = 5.928	*F*_1,28_ = 98.207
								*p* = 0.022	*p* < 0.001
								*η*_p_^2^ = 0.175	*η*_p_^2^ = 0.778
Step length (affected side, cm)	38.9 ± 5.6	42.1 ± 6.4^a^	3.2 (2.3, 4.2)^b^	39.6 ± 4.7	40.9 ± 4.8^a^	1.3 (1.0, 1.6)	0.14 (−0.58, 0.85)	*F*_1,28_ = 13.931	*F*_1,28_ = 78.315
								*p* = 0.001	*p* < 0.001
								*η*_p_^2^ = 0.333	*η*_p_^2^ = 0.737
Cadence (step/min)	80.5 ± 18.3	84.0 ± 23.3	3.5 (−1.6, 9.7)	80.1 ± 10.7	82.8 ± 11.0	2.7 (1.1, 4.3)	0.36 (−0.36, 1.08)	*F*_1,28_ = 0.070	*F*_1,28_ = 4.047
								*p* = 0.793	*p* = 0.054
								*η*_p_^2^ = 0.002	*η*_p_^2^ = 0.126
Under DT condition (performed with verbal fluency task)									
Speed (cm/sec)	55.1 ± 14.7	63.8 ± 15.6^a^	8.7 (7.3, 10.3)	54.9 ± 15.6	61.3 ± 19.2^a^	6.5 (4.2, 9.4)	0.06 (−0.66, 0.77)	*F*_1,28_ = 1.930	*F*_1,28_ = 89.731
								*p* = 0.176	*p* < 0.001
								*η*_p_^2^ = 0.064	*η*_p_^2^ = 0.762
TUG (sec)	23.6 ± 3.0	18.9 ± 3.1^a^	−4.7 (−6.0, −3.5)^b^	23.5 ± 4.8	21.0 ± 5.4^a^	−2.6 (−3.7, −1.4)	0.95 (0.19, 1.70)	*F*_1,28_ = 5.387	*F*_1,28_ = 64.125
								*p* = 0.028	*p* < 0.001
								*η*_p_^2^ = 0.161	*η*_p_^2^ = 0.696
Step length (affected side, cm)	40.2 ± 4.5	42.9 ± 4.8^a^	2.7 (1.9, 3.5)	39.6 ± 4.7	41.4 ± 4.4^a^	1.8 (1.4, 2.3)	0.56 (−0.17, 1.29)	*F*_1,28_ = 3.691	*F*_1,28_ = 98.044
								*p* = 0.065	*p* < 0.001
								*η*_p_^2^ = 0.116	*η*_p_^2^ = 0.778
Cadence (step/min)	80.0 ± 14.6	84.2 ± 15.1^a^	4.1 (2.6, 6.0)	81.8 ± 20.3	83.6 ± 20.0	1.8 (−1.7, 5.4)	0.16 (−0.56, 0.87)	*F*_1,28_ = 1.291	*F*_1,28_ = 8.198
								*p* = 0.266	*p* = 0.008
								*η*_p_^2^ = 0.044	*η*_p_^2^ = 0.226

*Note:* Mean ± SD presented for continuous variables; *η*_p_^2^: partial eta square.

Abbreviations: CI: confidence interval; S3S: serial‐3‐subtraction; VF: verbal fluency.

^a^ *p* < 0.05, significant within‐group difference.

^b^ *p* < 0.05, significant between‐group difference in change score.

**TABLE 3:** Performance of correct response rate (CRR).

	rTMS group			Control group			Effect size	Group × Time interaction effect	Time effect
	Pre	Post	Mean change after intervention (95% CI)	Pre	Post	Mean change after intervention (95% CI)	Hedges' *g* (95% CI)	F‐value, *p*‐value, *η*_p_^2^‐value	F‐value, *p*‐value, *η*_p_^2^‐value
Under ST condition									
S3S	0.21 ± 0.13	0.22 ± 0.15	0.01 (−0.01, 0.04)	0.20 ± 0.08	0.20 ± 0.11	0.00 (−0.03, 0.02)	0.19 (−0.53, 0.90)	*F*_1,28_ = 0.264	*F*_1,28_ = 0.732
								*p* = 0.612	*p* = 0.399
								*η*_p_^2^ = 0.009	*η*_p_^2^ = 0.025
VF	0.25 ± 0.09	0.27 ± 0.10	0.02 (−0.00, 0.05)	0.20 ± 0.08	0.23 ± 0.08^a^	0.03 (0.00, 0.06)	0.14 (−0.58, 0.86)	*F*_1,28_ = 0.127	*F*_1,28_ = 8.145
								*p* = 0.724	*p* = 0.008
								*η*_p_^2^ = 0.005	*η*_p_^2^ = 0.225
Under DT condition (performed with 10 m walking)									
S3S	0.19 ± 0.15	0.24 ± 0.16^a^	0.05 (0.02, 0.80)	0.18 ± 0.11	0.20 ± 0.12	0.02 (0.00, 0.04)	0.63 (−0.11, 1.35)	*F*_1,28_ = 2.947	*F*_1,28_ = 15.124
								*p* = 0.097	*p* = 0.001
								*η*_p_^2^ = 0.095	*η*_p_^2^ = 0.351
VF	0.22 ± 0.12	0.27 ± 0.13^a^	0.05 (0.04, 0.07)^b^	0.23 ± 0.16	0.24 ± 0.13	0.02 (−0.01, 0.04)	0.08 (−0.63, 0.8)	*F*_1,28_ = 5.079	*F*_1,28_ = 21.562
								*p* = 0.032	*p* < 0.001
								*η*_p_^2^ = 0.154	*η*_p_^2^ = 0.435
Under DT condition (performed with TUG test)									
S3S	0.14 ± 0.12	0.18 ± 0.12^a^	0.04 (0.02, 0.06)	0.14 ± 0.10	0.16 ± 0.11	0.02 (−0.00, 0.04)	0.75 (0.01, 1.49)	*F*_1,28_ = 2.570	*F*_1,28_ = 16.316
								*p* = 0.120	*p* < 0.001
								*η*_p_^2^ = 0.084	*η*_p_^2^ = 0.368
VF	0.15 ± 0.07	0.19 ± 0.08^a^	0.04 (0.02, 0.07)	0.14 ± 0.09	0.16 ± 0.11^a^	0.01 (−0.00, 0.03)	0.85 (0.10, 1.60)	*F*_1,28_ = 3.868	*F*_1,28_ = 15.227
								*p* = 0.059	*p* = 0.001
								*η*_p_^2^ = 0.121	*η*_p_^2^ = 0.352

*Note:* Mean ± SD presented for continuous variables, *η*_p_^2^: partial eta square.

Abbreviation: CRR: correct response rate (correct responses per second).

^a^ *p* < 0.05, significant within‐group difference.

^b^ *p* < 0.05, significant between‐group difference in change score.

**TABLE 4:** Cognitive and balance functions.

	rTMS group			Control group			Effect size	Group × Time interaction effect	Time effect
	Pre	Post	Mean change after intervention (95% CI)	Pre	Post	Mean change after intervention (95% CI)	Hedges' *g* (95% CI)	F‐value, *p*‐value, *η*_p_^2^‐value	F‐value, *p*‐value, *η*_p_^2^‐value
Cognitive function									
MoCA (0–30)	16.8 ± 5.3	23.9 ± 3.5^a^	7.1 (5.7, 8.6)^b^	16.9 ± 6.8	20.9 ± 7.4^a^	3.9 (1.6, 6.5)	0.79 (0.05, 1.54)	*F*_1,28_ = 4.687	*F*_1,28_ = 57.768
								*p* = 0.039	*p* < 0.001
								*η*_p_^2^ = 0.021	*η*_p_^2^ = 0.674
Stroop test									
Dots (s)	36.8 ± 21.0	30.4 ± 17.3^a^	−6.4 (−9.1, −4.5)	35.9 ± 18.1	30.6 ± 11.8^a^	−5.3 (−10.5, −1.7)	0.16 (−0.56, 0.87)	*F*_1,28_ = 0.180	*F*_1,28_ = 20.044
								*p* = 0.674	*p* < 0.001
								*η*_p_^2^ = 0.006	*η*_p_^2^ = 0.417
Words (s)	45.0 ± 28.5	39.6 ± 26.3^a^	−5.3 (−7.6, −3.3)	46.7 ± 18.3	42.6 ± 17.1^a^	−4.2 (−7.6, −1.1)	0.22 (−0.50, 0.94)	*F*_1,28_ = 0.360	*F*_1,28_ = 24.091
								*p* = 0.553	*p* < 0.001
								*η*_p_^2^ = 0.013	*η*_p_^2^ = 0.462
Colors (s)	64.6 ± 41.8	52.1 ± 28.3^a^	−12.5 (−22.5, −6.0)	65.8 ± 29.4	59.3 ± 28.1^a^	−6.47 (−8.7, −4.5)	0.50 (−0.23, 1.22)	*F*_1,28_ = 1.843	*F*_1,28_ = 18.277
								*p* = 0.185	*p* < 0.001
								*η*_p_^2^ = 0.062	*η*_p_^2^ = 0.395
TMT									
TMT‐A (s)	116.5 ± 78.1	90.6 ± 48.3^a^	−25.9 (−45.1, −11.8)	117.0 ± 72.7	98.2 ± 58.0	−18.8 (−58.2, 12.9)	0.13 (−0.58, 0.85)	*F*_1,28_ = 0.131	*F*_1,28_ = 5.286
								*p* = 0.720	*p* = 0.029
								*η*_p_^2^ = 0.005	*η*_p_^2^ = 0.159
TMT‐B (s)	235.1 ± 93.8	193.7 ± 98.9^a^	−41.5 (−75.8, −15.0)	227.8 ± 87.0	200.5 ± 103.0^a^	−27.3 (−57.8, −6.3)	0.26 (−0.46, 0.97)	*F*_1,28_ = 0.486	*F*_1,28_ = 11.529
								*p* = 0.491	*p* = 0.002
								*η*_p_^2^ = 0.017	*η*_p_^2^ = 0.292
Digit span test									
Forwards (2–8)	5.8 ± 1.7	6.7 ± 1.6^a^	0.9 (0.4, 1.4)	6.3 ± 1.5	6.7 ± 1.3	0.4 (−0.2, 1.0)	0.44 (−0.28, 1.17)	*F*_1,28_ = 1.460	*F*_1,28_ = 10.753
								*p* = 0.237	*p* = 0.003
								*η*_p_^2^ = 0.050	*η*_p_^2^ = 0.277
Backwards (2–8)	2.5 ± 0.9	3.3 ± 1.2^a^	0.8 (0.4, 1.2)	2.4 ± 1.6	2.8 ± 1.0	0.4 (−0.3, 1.1)	0.35 (−0.37, 1.07)	*F*_1,28_ = 0.933	*F*_1,28_ = 8.400
								*p* = 0.342	*p* = 0.007
								*η*_p_^2^ = 0.032	*η*_p_^2^ = 0.231
Balance									
Mini‐BESTest (0–28)	8.9 ± 4.9	15.0 ± 4.4^a^	6.1 (4.5, 7.6)	10.5 ± 5.9	13.9 ± 5.6^a^	3.3 (2.1, 4.8)	0.92 (0.17, 1.67)	*F*_1,28_ = 6.320	*F*_1,28_ = 74.741
								*p* = 0.018	*p* < 0.001
								*η*_p_^2^ = 0.184	*η*_p_^2^ = 0.727
ABC (%) (0–100)	59.6 ± 21.7	76.8 ± 18.2^a^	17.3 (10.8, 25.3)^b^	67.4 ± 19.0	72.3 ± 19.0^a^	4.9 (1.1, 8.0)	1.09 (0.33, 1.86)	*F*_1,28_ = 8.975	*F*_1,28_ = 28.809
								*p* = 0.006	*p* < 0.001
								*η*_p_^2^ = 0.243	*η*_p_^2^ = 0.507

*Note:* Mean ± SD presented for continuous variables; *η*_p_^2^: partial eta square.

Abbreviations: ABC: Activities‐specific Balance Confidence; Mini‐BESTest: Mini Balance Evaluation Systems Test; MoCA: Montreal Cognitive Assessment; TMT: Trail Making Test.

^a^ *p* < 0.05, significant within‐group difference.

^b^ p < 0.05, significant between‐group difference in change score.

### 3.2 Compliance and Adverse Events

The mean number of sessions attended for each participant was 9.2 ± 0.8 out of 10 sessions (92%), with no significant between‐group difference (*p =* 0.640). During the training period, no serious adverse events or mild adverse events (e.g., headache, scalp discomfort) were reported in either group during the 2‐week intervention.

### 3.3 Effect on Walking Ability Under ST Condition

All gait parameters (speed, cadence, and step length of the paretic leg) measured during the 10 m walking test and the time taken to complete the TUG test showed significant time effect (*p* < 0.001). Among these parameters, cadence (*F* = 8.272; *p* = 0.008; *η*_p_^2^ = 0.228) and step length of the paretic leg (*F* = 13.456; *p* = 0.001; *η*_p_^2^ = 0.325) during the 10 m walking test and TUG time showed significant group × time interaction effects (*p* < 0.01) (Table 2), indicating that the experimental group had better outcomes than controls (Hedges' *g* = 0.41–1.36) (Table 2).

### 3.4 Effect on Walking Ability Under DT Condition

Under DT condition with imposed serial 3 subtraction task, there was a significant time effect for speed, step length of the paretic leg and TUG time (*p* < 0.001). Cadence also showed a similar trend (*p* = 0.054) (Table 2). Significant group × time interaction effect was demonstrated in step length of the paretic leg (*F* = 13.931; *p* = 0.001; *η*_p_^2^ = 0.333) and TUG time (*F* = 5.928, *p* = 0.022, *η*_p_^2^ = 0.175), indicating that the experimental group had better outcomes than controls (Hedges' *g* = 0.14–0.91).

When the verbal fluency task was added, there were significant time effects on all gait parameters measured during 10‐m walk test and TUG time (*p* < 0.01) (Table 2). Significant group × time interaction effect was observed in TUG time (*F* = 5.387, *p* = 0.028, *η*_p_^2^ = 0.161) while that for step length of the paretic leg was marginal (*F* = 3.691, *p* = 0.065, *η*_p_^2^ = 0.116) (Hedges' *g* = 0.06–0.95).

### 3.5 Effect on CRR Under ST Condition

The CRR for the serial subtraction and verbal fluency task under ST condition did not show any significant group × time interaction effects (Table 3).

### 3.6 Effect on CRR Under DT Condition

When 10 m walking test was added, the CRR of both the serial subtraction and verbal fluency task showed significant time effect (*p* < 0.01). The group × time interaction effect was significant for the verbal fluency task (*F* = 5.079, *p* = 0.032, *η*_p_^2^ = 0.154), indicating that the experimental showed more improvement in CRR than controls (Hedges' *g* = 0.08). The serial 3 subtraction task also demonstrated a similar trend, although the result did not reach statistical significance (*F* = 2.947, *p* = 0.097, *η*_p_^2^ = 0.095).

For the TUG test under DT condition, the CRR also showed a significant time effect for both the serial 3 subtraction and verbal fluency task (*p* < 0.001). The experimental group had a greater improvement in CRR than controls (Hedges' *g* = 0.85), but the result did not quite reach statistical significance (group × time interaction effect: *F* = 3.868, *p* = 0.059, *η*_p_^2^ = 0.121) (Table 3).

### 3.7 Effect on Cognitive Function

Only MoCA scores showed both significant group × time interaction effect (*F* = 4.687, *p* = 0.039, *η*_p_^2^ = 0.021) with experimental group having better gain than controls (Hedges' *g* = 0.79). The other cognitive outcomes showed no significant group × time interaction effect, however (Table 4).

### 3.8 Effect on Balance and Balance Efficacy

Significant group × time interaction effects were found in Mini‐BESTest scores (*F* = 6.320; *p* = 0.018; *η*_p_^2^ = 0.184) and ABC scores (*F* = 8.975; *p* = 0.006; *η*_p_^2^ = 0.243), with the experimental group showing more improvement than controls (Hedges' *g* = 0.92–1.09) (Table 4).

## 4 Discussion

Findings of this study showed that rTMS to DLPFC combined with DT exercise training could augment the exercise training effect, compared with DT exercise training alone.

### 4.1 Intervention Effect on Walking Function

Goh et al. previously showed that one single session of 5 Hz rTMS applied to the left DLPFC did not induce any significant change in ST or DT walking speed among individuals with chronic stroke (Goh et al. 2020). On the other hand, our study showed that most gait parameters (cadence, step length of the paretic leg, and TUG time in ST condition; step length of the paretic leg and TUG time in DT conditions) showed significant improvements in the experimental group compared with the control group (Table 2, Hedges' *g* = 0.14–1.36).

The different findings between our study and Goh et al. may be caused by several reasons. First, we studied whether addition of rTMS to exercise training conferred additional therapeutic effect, while Goh et al. compared the effects between left DLPFC stimulation with M1 stimulation (site‐control, not sham‐control), and no exercise training was provided (Goh et al. 2020). Unlike Goh et al., who stimulated left DLPFC irrespective of lesion side, we targeted the ipsilesional DLPFC. rTMS may enhance DLPFC excitability, improving attentional allocation and reducing cognitive‐motor interference during dual‐task walking, thereby amplifying training‐induced neuroplasticity. Second, most participants in our study were at the sub‐acute stage (time since onset: 104.4 ± 25.5 days), which would have greater potential for recovery than that in Goh, et al. (time since onset: 22.8 ± 16.7 months). Lastly, only one single session intervention was applied in Goh et al., while our intervention protocol involved 10 consecutive sessions within a 2‐week period (Goh et al. 2020).

### 4.2 Intervention Effect on CRR Performance

Apart from the better gait performance after the experimental treatment, rTMS induced significantly better outcome in CRR performance when verbal fluency task was performed in conjunction with 10 m walk (Hedges' *g* = 0.08) (Table 3). In addition, there was a trend of better improvement in CRR in the experimental group for other DT conditions (Table 3, Hedges' *g* = 0.08–0.85). Taken together, stimulation of DLPFC enhanced the overall DT walking and cognitive performance, rather than a trade‐off between motor and cognitive function. A previous randomized controlled study in chronic stroke showed that 8 weeks of DT training led to improvement in DT walking speed but no change in CRR under DT conditions compared with controls (Pang et al. 2018). In this study, adding rTMS to DT exercise training may have additional benefit of improving cognitive performance under DT condition. DLPFC plays a critical role in the executive function and higher order cognitive function, Lattari et al. (2017) and Swank et al. (2016) and may thus be involved in mediating DT walking performance (Goh et al. 2020; P. L. Wong et al. 2022). Our results suggest that simulating this brain region using rTMS may facilitate better recovery of DT function in people with sub‐acute stroke.

### 4.3 Intervention Effect on Balance and Cognitive Functions

Among the various cognitive outcomes, MoCA scores showed greater improvement in the experimental group than control group. As identified in previous reviews and meta‐analysis, non‐invasive brain stimulation can effectively improve cognitive function in people with stroke (Hara et al. 2021; Begemann et al. 2020).

Other cognitive measures, including the Stroop test (attention), TMT‐A and B (executive function), and digit span test‐forward and backward (working memory) significantly improved over time in both groups. It is known that participation in physical exercise may also have positive effect on cognitive function post‐stroke (Draaisma et al. 2020). The participants were in the sub‐acute stage and thus the extent of improvement may be more prominent than later stages (Lugtmeijer et al. 2021). Perhaps a more intensive rTMS protocol is required to induce between‐group differences in these cognitive outcomes.

The balance function (Mini‐BEST score) and balance efficacy (ABC score) were all significantly improved over time, indicating that DT exercise may be beneficial in improving these outcomes for individuals at this stage. In addition, the experimental group had greater gain in these two outcomes than controls, further demonstrating that adding rTMS can augment the exercise training effect. The better ST and DT walking ability as well as overall balance function in the experimental group may contribute to greater gain in balance efficacy.

### 4.4 Limitations and Future Research Directions

The findings of our study are only generalizable to individuals with sub‐acute stroke who have mild to moderate motor and cognitive impairment. DLPFC localization was based on surface anatomy (the “5 cm rule”), which is less precise than MRI‐guided neuronavigation. This may have introduced variability in stimulation accuracy and is a limitation of our study. We did not formally assess blinding efficacy. However, all participants were told that the rTMS sensation would be minimal, and the sham group received an identical coil placement and sound. Future studies should include a blinding questionnaire. Due to concerns with potential physical and mental fatigue of participants during testing, only two cognitive tasks (serial subtraction, verbal fluency) were used in the DT testing paradigm, while other cognitive tasks (e.g., working memory, reaction time) were not tested. Future study should include working memory and reaction time tasks to evaluate broader cognitive effects. No follow‐up assessment was conducted; therefore, the durability of treatment effects beyond 2 weeks remains unknown. Future trials should include a longer follow‐up period. In addition, future studies should incorporate functional neuroimaging (e.g., functional near infrared spectroscopy, etc.) to confirm whether rTMS‐induced DLPFC activation reduces prefrontal overactivation during DT walking—a common compensatory pattern post‐stroke.

## 5 Conclusion

The 2‐week rTMS to DLPFC combined with DT exercise training augments DT walking performance, and improves balance and cognitive function in individuals with sub‐acute stroke who have mild to moderate motor and cognitive impairment.

### 5.1 Implications of Physiotherapy Practice

This combined intervention can be implemented in inpatient stroke rehabilitation in the sub‐acute stage: rTMS before daily dual‐task physiotherapy for 2 weeks is feasible and well‐tolerated.

## Author Contributions

L.Y., X.L., and M.Y.C.P. to study conception. X.L., J.L., X.C., L.W., H.Y., and Y. G. to data acquisition. L.Y., X.L. to data collection. L.Y., and M.Y.C.P. to data analysis and interpretation. L.Y., and M.Y.C.P. to manuscript writing. All authors read and approved the final version of the manuscript.

## Funding

Kunming Health Science and Technology Talent Training Project, Training Plan for Medical Science and Technology Discipline Leaders (No. 2022‐SW [Leaders]‐27), Yunnan Province, China; and Chuncheng Project, The cultivation of top talents, The program of Chuncheng prestigious doctors (Grant C202212011), Yunnan Province, China.

## Conflicts of Interest

The authors declare no conflicts of interest.

## Data Availability

The data associated with the paper are not publicly available but are available from the first author on reasonable request.
